# Supplementary material for: Resolving the full spectrum of human genome variation using Linked-Reads
Source: Genome Res. 2019 Apr;29(4):635–45. doi: 10.1101/gr.234443.118 (PMC6442396; doi:10.1101/gr.234443.118)
Supplement: Supplemental Material [file supp_gr.234443.118_Supplemental_longranger-2.2.zip › longranger-2.2.tar.bz2!/lr2.2-public/lib/stan/cmdstan/stan/doc/api/html/math_2matrix_2divide_8hpp_source.html]

Stan: src/stan/math/matrix/divide.hpp Source File


|  |  |
| --- | --- |
|  | Stan  2.5.0  probability, sampling & optimization |


- Main Page
- Related Pages
- Namespaces
- Classes
- Files

- File List
- File Members

All Classes Namespaces Files Functions Variables Typedefs Enumerations Enumerator Friends Macros Pages

- src
- stan
- math
- matrix

divide.hpp

Go to the documentation of this file.

1 #ifndef STAN\_\_MATH\_\_MATRIX\_\_DIVIDE\_HPP

2 #define STAN\_\_MATH\_\_MATRIX\_\_DIVIDE\_HPP

3

4 #include <boost/type\_traits/is\_arithmetic.hpp>

5 #include <boost/utility/enable\_if.hpp>

6 #include <stan/math/matrix/Eigen.hpp>

7

8 namespace stan {

9  namespace math {

10

19  template <int R, int C, typename T>

20  inline

21  typename boost::enable\_if\_c<boost::is\_arithmetic<T>::value,

22  Eigen::Matrix<double, R, C> >::type

23  divide(const Eigen::Matrix<double,R,C>& m,

24  T c) {

25  return m / c;

26  }

27

28  }

29 }

30 #endif

Eigen.hpp

stan::math::divide

int divide(const int x, const int y)

**Definition:** divide.hpp:11


---

[ Stan Home Page ]

*© 2011–2014,
Stan Development Team.*
